# Genetic evidence for plastic reproductive philopatry and matrotrophy in blacktip reef sharks (*Carcharhinus melanopterus*) of the Moorea Island (French Polynesia)

**DOI:** 10.1038/s41598-023-40140-6

**Published:** 2023-09-09

**Authors:** Kim B. Eustache, Émilie Boissin, Céline Tardy, Ian A. Bouyoucos, Jodie L. Rummer, Serge Planes

**Affiliations:** 1 PSL Research University, EPHE-UPVD-CNRS, UAR 3278 CRIOBE, Université de Perpignan, 58 Avenue Paul Alduy, 66860 Perpignan Cedex, France; 2 Institute for Biodiversity and Ecosystem Dynamics, University of Amsterdam, Amsterdam, The Netherlands; 3 Laboratoire d’Excellence “CORAIL”, Papetoai, Moorea, French Polynesia; 4 WWF-France, 6 rue des Fabres, 13001 Marseille, France; 5 Department of Biological Sciences, University of Manitoba, Winnipeg, MB R3T 2N2 Canada; 6 Australian Research Council Centre of Excellence for Coral Reef Studies and College of Science and Engineering, James Cook University, Townsville, QLD Australia

**Keywords:** Ecology, Evolution, Genetics

## Abstract

The exploitation of sharks and the degradation of their habitats elevate the urgency to understand the factors that influence offspring survival and ultimately shark reproductive success. We monitored and sampled blacktip reef sharks (*Carcharhinus melanopterus*) in nursery habitats of Moorea Island (French Polynesia), to improve knowledge on shark reproductive behavior and biology. We sampled fin clips and morphometrics from 230 young-of-the-year sharks and used microsatellite DNA markers to process parentage analysis to study the reproductive philopatric behavior in female sharks and the matrotrophy within litters. These traits are driving the success of the local replenishment influencing selection through birth site and maternal reserves transmitted to pups. Parentage analysis revealed that some female sharks changed their parturition areas (inter-seasonally) while other female sharks came back to the same site for parturition, providing evidence for a plastic philopatric behavior. Morphometrics showed that there was no significant relationship between body condition indices and nursery locations. However, similarities and differences in body condition were observed between individuals sharing the same mother, indicating that resource allocation within some shark litters might be unbalanced. Our findings further our understanding of the reproductive biology and behavior that shape shark populations with the aim to introduce these parameters into future conservation strategies.

## Introduction

Reef sharks are mesopredators on coral reef ecosystems in tropical regions of all oceans^1^. They play a key role in coral reef food webs through the consumption of preys influencing the prey’s behavior^2^. Reef sharks are subject to increasing fishing pressure and disturbances on a global scale^3–6^. Their habitats are often located near coastlines making them vulnerable to human activities and impacts^7^. Identifying reef shark habitats and understanding their reproductive behavior has become an urgent need to implement effective conservation strategies, targeting specific habitats^8–10^.

Blacktip reef sharks (*Carcharhinus melanopterus*) were listed as vulnerable on the IUCN Red List of Threatened Species in 2020 under the criteria A2bcd^11^. Their life history strategy with slow growth (mean growth rate is c. 6 cm.year^−1^^10^), a late sexual maturity (e.g., approximately 4 years for males and 8 years for females in blacktip reef sharks) and a high young of the year (hereafter referred to as “juvenile”) mortality rate^4,12,13^ makes this species even more vulnerable to any kind of continuous exploitation. Females of blacktip reef sharks give birth in shallow coastal habitats that are identified to provide a safe environment against predation, with sufficient resources for their pups^10^. However, in coastal seascapes juvenile sharks are exposed to physical perturbations such as being part of bycatch in fishing activities (this can lead to deadly stress levels^14,15^), coastal infrastructure development (e.g., harbors, jetties, coastal embankments), and tourism expansion together with being exposed to material and sound pollution^16^. These disturbances may increase the already high natural mortality rates^16^. The juvenile mortality rate was estimated to be between 61 and 92% of the annual recruitment for *Carcharhinus limbatus* in coastal nurseries emphasizing the key role of nurseries in the life cycle and the demography of coastal sharks^13^.

Together with environmental features, females also influence the survival of their pups through maternal resource allocation (matrotrophy^17,18^). Females of blacktip reef sharks give birth to an average of 3 to 5 pups^19^ after a gestation period of 286 to 305 days^12^. Neonate sharks are characterized by enlarged livers that are used for lipid storage and as an energy resource during the first weeks following birth and during the transition (after about three weeks following birth) to an independent foraging capacity that will drive survival after the maternal lipid reserves are consumed^17,20,21^. The body condition of juvenile sharks might be variable within the litter, favoring some individuals by enabling them with a better survival capacity. Juvenile sharks that have higher lipid reserves might be able to endure longer starvation periods while acquiring hunting skills^17^. Variability in juvenile shark body condition might be attributed to the environmental variability or to the mother’s phenotype, depending on their age^22^. Until they have acquired efficient hunting and scavenging skills (after about five weeks after birth), their body condition is mostly maintained by the accumulated macromolecular resources in the liver initially provided by the mother^22^. Mothers might give birth to sharks that have the same body condition or to offspring with different body conditions within the same litter. Such knowledge is essential to set a basis for understanding how the condition of juveniles affects their survival. Some sharks might have a better condition at birth leading to higher survival chances independent of the nursery environment.

Previous studies on blacktip reef sharks from Moorea (French Polynesia) suggested female philopatry, as they often gave birth in the same locations over several successive birthing events. Such behavior combined with restricted movements and dispersal, can also result in high inbreeding levels amongst the population^10^. Philopatric behavior of female sharks contributes to reduce connectivity among populations (already isolated in the context of oceanic islands in French Polynesia) where intrinsic reproduction and recruitment become the determinants of population dynamics^23^. Philopatry in sharks is a double-edged sword likely increasing the adaptation across heterogenous environments, the fitness by providing predictable access to food and shelter from predators but limiting the gene-flow because of the isolated oceanic islands context^10,24^. Moorea is a Polynesian island that has different types of ecosystems along its coastline including mangroves, sandflats, and coral reefs, which may influence the population dynamics of the local shark populations as sharks show complex habitat use patterns^3^.

The objective of this study was to retrieve behavioral and biological patterns in female blacktip reef shark reproduction from the two-years monitoring of juvenile sharks inhabiting nursery habitats of the Moorea Island (French Polynesia). We investigated whether some adult females would display a reproductive philopatric behavior and if there was a disparity in the matrotrophy within litters. Parentage analyses were used to identify the interannual full-siblings to search for reproductive philopatry, the fidelity of mother sharks to their parturition areas. Body condition was used as a proxy for maternal investment, which allowed us to determine if there was variability in body condition between full-siblings (individuals of the same year, having the same mother).

## Results

**Figure 1:**
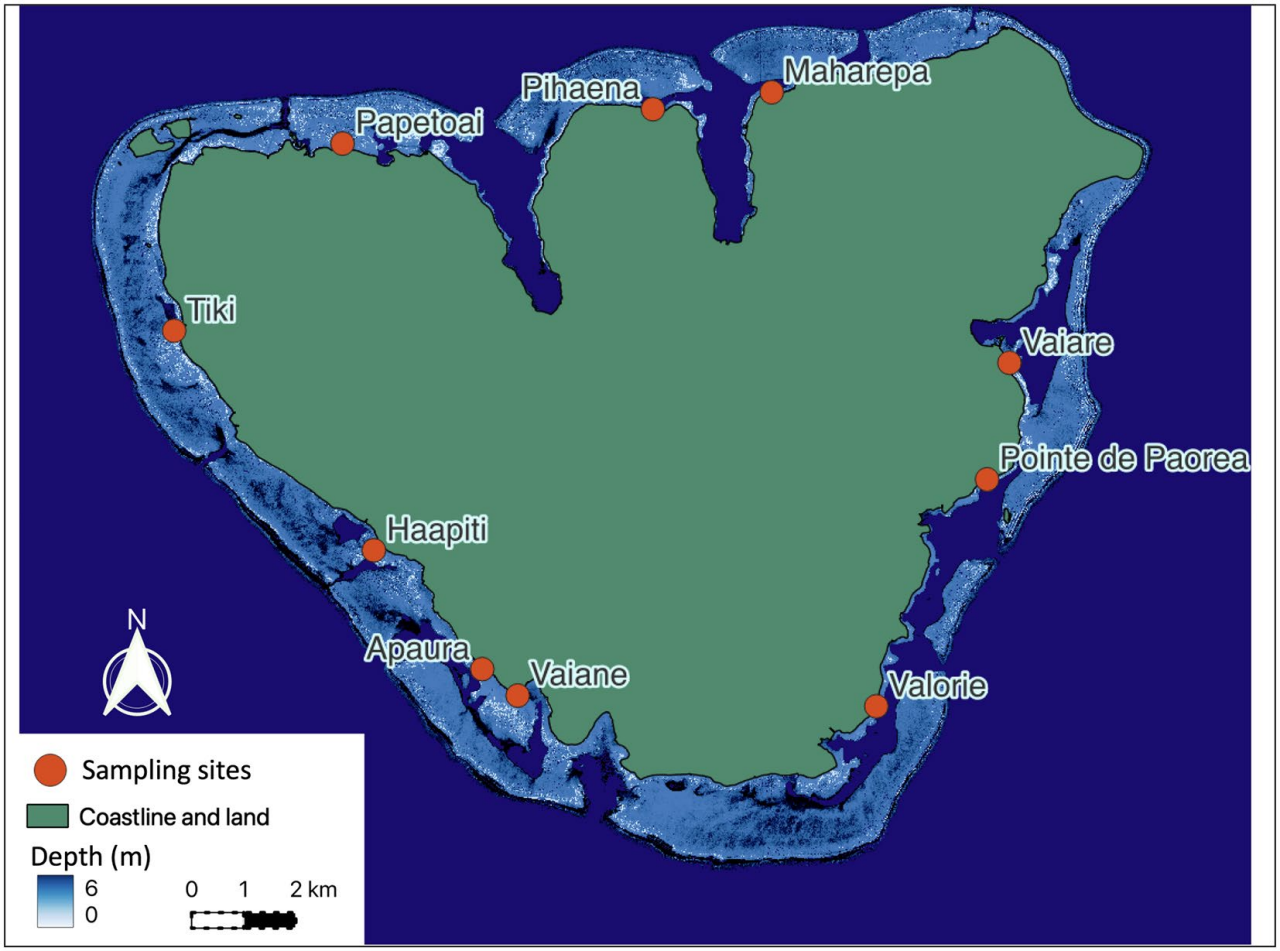
Map of the ten sampling sites of Moorea Island (French Polynesia) created using QGIS v.3.32.0, URL: https://timdocs.qgis.org/en/site/index.html.

A total of 230 juvenile blacktip reef sharks were collected from October until the end of Ferbruary in 2017–2018 and 2018–2019 during a total of 171 sampling events from ten sampling sites around Moorea Island (Fig. 1). A total of 136 juveniles were sampled in 2017–2018 and 94 in 2018–2019.

### Parentage analyses

Parentage analyses per season for each individual site were cross-validated with all sampled individuals and revealed four pairs of full-siblings in two locations, Pointe de Paorea in 2017–2018 and Haapiti in 2018–2019 (Table 1). Both sites also resulted in the highest number of sampled sharks for their respective season. Half-siblings were present at all locations, with the highest number identified at Maharepa and Haapiti in 2017–2018, and at Papetoai and Haapiti in 2018–2019.

**Table 1:** Summary of the parentage analyses: number of individuals per site (N) for the per site analysis (the results were validated by running the entire dataset both within years and across years), for the sampling season 2017–2018 (S1), the sampling season 2018–2019 (S2), and inter-annually (S1&S2).

Location	Season	*N*	Pointe de Paorea		Maharepa		Haapiti		Tiki		Papetoai		Vaiare		Valorie	
FS/HS			FS	HS	FS	HS	FS	HS	FS	HS	FS	HS	FS	HS	FS	HS
Pointe de Paorea	S1	36	2	9											Nd	Nd
	S2	10		1	Nd	Nd			Nd	Nd						
	S1&S2	230	2				1							7		
Maharepa	S1	26				14									Nd	Nd
	S2	2	Nd	Nd	Nd	Nd	Nd	Nd	Nd	Nd	Nd	Nd	Nd	Nd	Nd	Nd
	S1&S2	230														
Haapiti	S1	24						10							Nd	Nd
	S2	32			Nd	Nd	2	5	Nd	Nd						
	S1&S2	230					1	11								
Tiki	S1	17								1					Nd	Nd
	S2	4	Nd	Nd	Nd	Nd	Nd	Nd	Nd	Nd	Nd	Nd	Nd	Nd	Nd	Nd
	S1&S2	230					1									
Papetoai	S1	11										8			Nd	Nd
	S2	19			Nd	Nd			Nd	Nd		9				
	S1&S2	230					1					14				
Vaiare	S1	13												2	Nd	Nd
	S2	9			Nd	Nd			Nd	Nd				2		
	S1&S2	230											1	1		
Valorie	S1	0	Nd	Nd	Nd	Nd	Nd	Nd	Nd	Nd	Nd	Nd	Nd	Nd	Nd	Nd
	S2	12			Nd	Nd			Nd	Nd						2
	S1&S2	NA														

The table indicates the results of the parentage analysis (for parental links with a posterior probability > 0.75) per sampling season for the pairs of full-siblings (FS) and pairs of half-siblings (HS). The cells with “Nd” (i.e., not determined) indicate which analysis could not be performed because of an insufficient sampling size per site (N ≤ 4).

We explored sibs among sampling seasons (from October to February) within and between nurseries using parentage analysis. Regarding inter-seasonal relationships (one individual caught during the sampling season of 2017–2018 and one individual caught during the sampling season 2018–2019) within nurseries, one pair of inter-seasonal full-siblings (COLONY, N = 56 [S1 + S2: total number of sharks used in the inter-seasonal relatedness analysis]) (Table 1, *P* = 0.765) and 11 pairs of inter-annual half-siblings were found in Haapiti (N = 56 [Table 1, *P* = 0.784 ± 0.055]). We found two pairs of inter-seasonal full-siblings for Pointe de Paorea (N = 46 [Table 1], *P* = 0.775 ± 0.009), and one pair of inter-seasonal full-siblings for Haapiti (N = 153, *P* = 0.856) and one pair of inter-seasonal half-siblings for the site Vaiare (N = 21, *P* = 0.701). Regarding inter-seasonal relationships between nurseries, one pair of full-siblings were identified between Pointe de Paorea (2017–2018) and Haapiti (2018–2019) (N = 153, *P* = 0.798). One pair between the sites Haapiti (2017–2018) and Papetoai (2018–2019) (N = 153, *P* = 0.825) was found. Finally, one pair of inter-seasonal full-siblings were found between the sites Tiki and Haapiti (N = 153, *P* = 0.871).

### Differences in the body condition of shark litters

**Figure 2:**
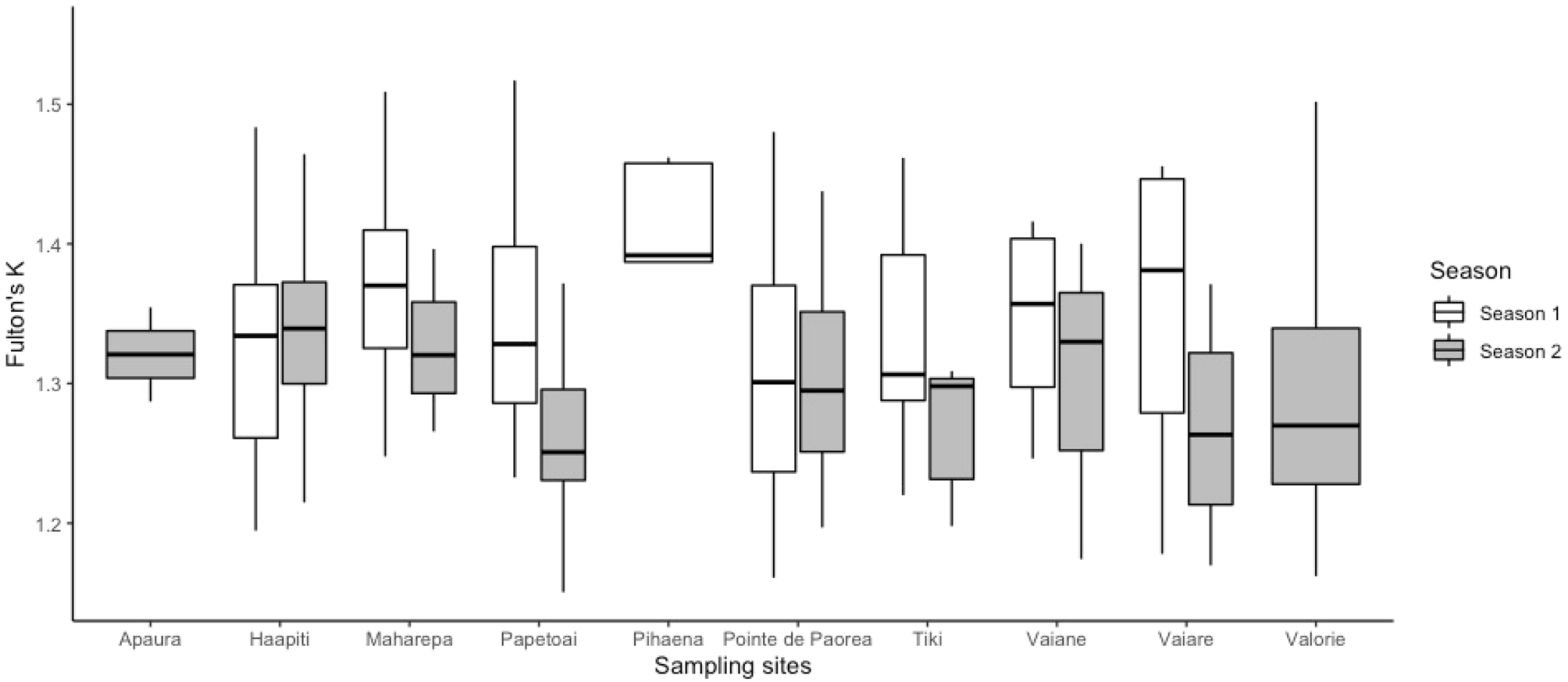
Mean and 95% confidence interval of Fulton’s K index per sampling site for the sampling season 2017–2018 (Season 1) and for the sampling season 2018–2019 (Season 2). The sites Apaura, Pihaena and Valorie have larger boxplots as they were sampled in a single season.

Fulton’s K, across all sites during 2017–2018, was 1.3 ± 0.19, and there were no significant differences in K between sites (one-way ANOVA, F_(1, 128)_ = 0.827, *p* = 0.567; Fig. 2). There were cases of differing K between full-siblings, however. At Pointe de Paorea, for example, one set of full-siblings caught on the same day had the same pre-caudal length (44 cm) but different body masses (1.2 and 1.0 kg), thus resulting in a higher K for the female (i.e., 1.42) than for the male (i.e., and 1.17). At Pointe de Paorea a pair of full-siblings had similar K indexes (female, K = 1.32; male, K = 1.32) and were caught during the same sampling event.

Fulton’s K, across all sites during 2018–2019, was 1.29 ± 0.08 and there were no significant differences in K between sites (one-way ANOVA, F_(1, 93)_ = 1.443 *p* = 0.189; Fig. 2). There were multiple cases of full-siblings (COLONY, *P* = 0.92 ± 0.076), but at Haapiti, neither the first pair that were caught in the same sampling event (female, K = 1.3; female, K = 1.31) nor the second pair that were caught in different sampling events (male, K = 1.33; female, K = 1.32) exhibited differences in K. Overall, our analyses revealed that three pairs of full-siblings presented almost similar K indices (average K indices and standard deviation 1.31 ± 0.01), and one pair of full-siblings presented different K indices (1.29 ± 0.17).

## Discussion

The objective of this study was to explore reproductive philopatry in female sharks and the matrotrophy among litters. Full-siblings, juvenile shark individuals having the same mother and father, were found in some nurseries as well as between nurseries within the same sampling season, and between the two sampling seasons, thus providing a glimpse at female shark reproductive strategies. Matrotrophy is likely to be variable among litters as three pairs of full-siblings showed within-pair morphometric similarities and one pair displayed morphometric variability.

The parentage analysis showed that several juvenile sharks caught in the same sites over the two seasons had the same parents, which suggests that mothers came back during two seasons to the same area to give birth (i.e., philopatry). Full-siblings occurred at Haapiti and Vaiare, and half-siblings occurred at Papetoai and Pointe de Paorea. In the latter instance, whilst the assumption was that they shared the same mother, the common parent could only be validated with certainty for full-sibling pairs. First, these results confirm some annual breeding events in female blacktip reef sharks in Moorea as suggested earlier in Mourier and Planes^10^. Moreover, inter-annual full-siblings indicate chosen or forced fidelity to the same mating partner, either by females choosing the same sires, by males choosing the same females, by dominant males annihilating the competition for females, or by sperm storage^25,26^. Although, sperm storage has been described in other Carcharhinid species, it is unknown whether it occurs in blacktip reef sharks^27,28^. Inter-annual full-siblings also imply female breeding site philopatry, which was previously suggested for Haapiti, as three interannual full-sibling pairs were recovered in an earlier relatedness analysis^10^. However, philopatry might be a non-exclusive behavior of females in this species, as full-siblings were also found between potential nursery sites located on opposite sides of the island; Pointe de Paorea (2017–2018) and Haapiti (2018–2019) and between Haapiti (2017–2018) and Papetoai (2018–2019). We demonstrate here that females do not give birth exclusively in the same nurseries (plastic reproductive philopatry) and that females randomly give birth in the different potential nurseries around the island. These findings do not confirm the (exclusive) philopatry hypothesis for this species^10^, or that their reproductive behavior could have been altered by human impact. For the latter explanation, anthropogenic perturbations, such as overfishing, coral coverage loss^29,30^, or climate change^14^ could force mothers to give birth in other areas. This has been the case for philopatric nesting marine turtles, as their reproduction behavior has been altered by coastal development and artificial lighting^31^. Similar interruptions have been documented in sea birds, teleost fishes, and marine mammals, which has prompted the importance of including such species-specific reproductive areas in management plans to decrease the impact of human activities in situ^32^. In the case of blacktip reef sharks in Moorea, the dramatic increase in human population and activities that has occurred over the last decade^33^ might have disrupted the inter-generational female philopatric reproduction behavior. Such findings highlight the plasticity in the reproductive behavior of female sharks either randomly giving birth in different nursery areas or changing their parturition areas upon disturbances in their preferred site. The plasticity in female philopatric reproductive behavior is likely derived from the unsystematic utilization of nursery grounds specific to each individual, in addition to their adaptability to local (anthropogenic) disturbances^34^.

The variability in body condition (i.e., Fulton’s K index) between the sampling sites and seasons was not significant, and we found similarities in three pairs of full-siblings and differences in one full-sibling pair. There were no significant differences among sites in the K index neither for the sampling season 2017–2018 nor for the 2018–2019 season. As the body conditions of juvenile sharks, combined, seem to be independent from habitat (i.e., mangroves, sand flats, coral covered areas) and birth location, maternal effects—such as matrotrophy, or the continuous extra-vitelline supply of nutrients from the parent to the progeny during gestation^35^ may be critical.

Our original combination of relatedness and body condition (K) analysis revealed similarities and differences in individuals having the same mother. Indeed, the descriptive analysis showed that one pair of full-siblings had greater differences in K than the three other pairs of full-siblings. The pair of full-siblings having the highest difference in their K index was one pair of juvenile sharks caught the same day, and this difference was driven by body mass, as both sharks had the same length. The differences in body condition are most likely due to maternal investment or foraging capacity and available resources. The liver of juveniles of carcharhinid species represents at birth between 10 and 20% of the total body mass and acts as a storage for lipid reserves, a maternal head-start that provides sufficient energy to reach the independent foraging state^17,18,20^. Moreover, juvenile sharks are known to lose weight after depleting those liver lipid reserves in the first few weeks after birth^18,20^. The three pairs of full-siblings that had little differences in body condition might indicate that maternal reserves are in general evenly distributed amongst the litters of blacktip reef sharks. Recent studies uncovered that the body condition of sharks decreases after about five weeks in synchronicity with the moment they start exploring their surroundings to find food resources^37^. Three pairs of full-siblings were captured together in the same locations on the same day. This finding supports the hypothesis proposed in previous studies for this species of sharks, suggesting that siblings display companionship behavior by staying together during their early weeks of life^36^. Social networks among adult sharks, on the other hand, were found to be unrelated to kinship, likely due to the dispersion of siblings after five weeks^38^. The observed companionship among shark pups in nursery areas may indicate that these pups are younger than five weeks old, as they seem to be conserving energy and not yet exploring their surroundings, possibly relying on their liver reserves. However, further research is needed to determine the age of juvenile sharks using emerging methodologies^37^. This exploratory data provides only preliminary insights into blacktip reef shark matrotrophy but more data on full-siblings is required to draw quantitatively-based conclusions regarding the difference in maternal investment within litters and among mothers.

Our findings have consequences for the management of blacktip reef sharks, a widely distributed shark species present on most tropical coral reef ecosystems. Our innovative method, combining analyses of relatedness and body condition factor that proved to be successful at studying matrotrophy in sharks encourages further data collection to draw more solid patterns among female sharks and their litters. The evidence found for non-exclusive reproductive philopatric behavior, highlights plasticity in female reproductive behavior thus enhancing their capacity to adapt to environmental changes (e.g., local anthropogenic disturbances, climate change) while securing local replenishment.

## Material and methods

### Location and sampling

All shark capture and research protocols were approved under Arrêté N° 9524 issued by the Ministère de la Promotion des Langues, de la Culture, de la Communication et de l’Environnement of the French Polynesian government on 30 October 2015 and James Cook University’s Animal Ethics Committee (A2394 and A2769). All methods were performed in accordance with the guidelines and regulations provided by the French Polynesian government and the James Cook University’s Animal Ethics Committee. Around the island of Moorea, (French Polynesia, Fig. 1), 10 sites (Apaura, Haapiti, Maharepa, Paorea, Papetoai, Pihaena, Tiki, Vaiane, Vaiare, and Valorie), have been annually monitored and sampled during parturition seasons (from October to February each year) as part of long-term population dynamic surveys carried out by the Centre de Recherches Insulaires et Observatoire de l’Environnement (CRIOBE), as these 10 sites have been identified as inshore reef shark nursery areas^10,35^ (Fig. 1).

Typically, all sites are sampled twice per month between October and February every year^34^. Apaura and Valorie could not be sampled at all during the 2017–2018 season. For the second season, 2018–2019, sampling started in October 2018 and concluded at the end of February 2019 for all sites except Vaiane, Vaiare, and Valorie, where sampling ended at the end of January 2019 (Fig. 1).

Sharks were caught using a 50 m × 1.5 m gillnet with a 5 cm mesh size that was set perpendicular to the shoreline between the hours of approximately 5 pm to 8 pm each evening. When a shark was caught, it was immediately removed from the net, tagged with a passive integrated transponder (PIT, providing a unique ID code), fin-clipped, sexed, measured with several morphometrics (cm), weighed (kg), and the umbilical scar was photographed with a size reference. Neonatal sharks of this species have open umbilical scars, which indicates they were born less than 30 days ago. The entire procedure lasted less than 10 min and was designed to minimize the stress caused to the animals through rapid interventions. While the PIT tags and fin clips caused lesions, the fast-healing capacities of sharks allows them a complete healing within a few days for the lesion caused by the PIT tag^36,37,39^ and about 7 months for the end of the dorsal fin to fully grow back (personal observation). Fin clips were stored in 1.5 mL microtubes filled with 95% ethanol and preserved for subsequent genetic analysis.

### Body condition factor

The Fulton’s K body condition index was calculated for the juvenile shark individuals caught during the two sampling seasons using the following equation: K = 10^5^M_TB_(L_PC_^3^)^−1^^18,38^, where M_TB_ is the total body mass (g) and L_PC_ is the total pre-caudal length (cm). As the data were normally distributed, a one-way ANOVA (95% confidence interval) was used to identify differences in K within each sampling season (2017–2018 and 2018–2019) and between sampling sites. Then, pairs of full-siblings from the same sampling season (to make sure they originate from the same litter) were assigned their sex, date of capture, and K to provide insight as to the body condition of neonate and young of the year juvenile blacktip reef sharks within the same litter. When applicable, results were reported as means ± SD when summarizing the results of K of several sharks.

### Molecular analyses

Total DNA was extracted from each fin clip with the QIAcube HT (© QIAGEN, Hilden, Germany) DNA extraction robot. After DNA extraction, three multiplex polymerase chain reactions (PCRs) were performed per individual, using fluorescently labelled primers to screen 14 microsatellite loci (Table S1). Microsatellite markers were retrieved from^10^ but were initially developed from previous studies (i.e.,^44–48^; Table S1). PCRs were realized in 10μL containing 4μL of QIAGEN Type-it Microsatellite PCR kit, 4 μL of RNase-free water (provided with the kit), and 1.9 μL of DNA template 40 ng/µL. The primers were added in different amounts, depending on the previous optimization tests and adaptations of the quantities, with 0.01 μL of each primer (100 μM). Two multiplexes of 5 loci and one multiplex of 4 loci were run for all 230 samples. Amplifications were carried out as follows: 5 min at 95 °C, followed by 45 cycles of 30 s at 95 °C, 1 min 30 s at optimal annealing temperature (57–63 °C, depending on the locus, Table S1), 30 s at 72 °C and a final extension of 30 min at 60 °C. PCR products were then run on 1.5% agarose gels to check for correct amplifications. PCR products were sent to a private company, GenoScreen (Lille, France), for subsequent genotyping. The resulting electropherograms were scored using GeneMapper v.5 software (Applied Biosystems)*.* The loci that were partially or completely unreadable were reamplified for each individual, sent again to GenoScreen, scored, and then compared with the former version of the electropherogram. Individuals that had less than 70% of their genotype were removed from the study. The software MICRO-CHECKER v.2.2.3^40^ was used to search for the presence of null alleles, scoring errors, and large allele dropouts as the number of samples was sufficient (genetic summary statistics available in Table S1).

### Data analyses

#### Parentage analysis

Deviations from the Hardy–Weinberg equilibrium were estimated for each locus using GENEPOP online v.4.6^41^ and for each sampled site using GENETIX v.4.05^42^ (Table S1). Parental links between individuals were investigated using COLONY v.2.0.6.5^43^. This computer program implements maximum likelihood methods to estimate full and half-sibling relationships among multi-locus genotyped individuals^43^. COLONY listed all relationships that were not excluded as true relationships with a 95% confidence interval^43^. The analysis parameters were set up using a mating system with female and male polygamy with the possibility of inbreeding but without clones. The analysis method was made of 3 runs set as ‘long’ runs, with full likelihood and a likelihood precision that was set to ‘high’. Then, a more limiting criterion was implemented by validating only the relationships that were assigned a posterior probability (*P*) ≥ 0.75 or higher and that were identified in all three runs^44^. The results were written as means ± SD for reporting multiple posterior probabilities. We favored a conservative approach that might have underestimated the number of individuals per family but minimized the false positive parentage assessments^44^. Two types of analyses were run to investigate relationships, (i) within nurseries with a cross-validation investigating parental relationships with all samples per sampling season and (ii) between sampling seasons with the samples from both seasons and all sites analyzed together. The percentages of recaptures per season and per site were calculated as well as the percentages of recaptures for both seasons combined but per site. The results of the per-season and inter-season parentage analyses were used to explore the female reproductive and juvenile behavior.

#### Within nursery relationships

The individuals captured in the same nursery during the same sampling period were analyzed together for parentage inference for each of the two sampling years separately. Afterward, all individuals from one sampling season were analyzed together to evaluate the robustness of the parentage relationships found with the per-sampling site parentage analysis. Sites with N ≤ 4 were not analyzed individually for parental relationships, as it was considered that such low sample sizes could not represent the total number of neonate sharks born during that year within a particular area of the island decreasing the robustness of parentage analysis. Indeed, the focus of this study was to look for individuals that had the same parents without retracing the individual parents. The objective was to identify full-siblings to search for connectivity between the nurseries, redundancy of attendance in the parturition behavior of females, and variance in the body condition within a litter. The individuals from the sites that were not analyzed individually, because of the low sample size, were used in cross-validation analyses with all individuals from the sampling season. For the sampling year 2017–2018, fewer than four sharks were sampled from Vaiane, Apaura, Valorie, and Pihaena and were thus not analyzed individually for parentage. Similarly, for the sampling year 2018–2019, fewer than four sharks were caught from Tiki, Vaiane, Maharepa, Pihaena, and Apaura, and so they were not analyzed individually for parentage.

#### Reproductive strategy

Parentage analysis was thus used to search for inter-annual relationships between the juvenile sharks that were caught in the same nurseries. Indeed, inter-annual full-siblings caught in the same sites have the same mother. Sites, where more than four individuals were caught for both seasons, were analyzed together for parentage. Then, the sites were analyzed for parentage altogether across both seasons. This allowed us to search for inter-annual parentage within and between the sites for Pointe de Paorea, Haapiti, Papetoai, and Vaiare because they had sufficient juveniles over two seasons for analysis and to determine whether the female returns to the same nursery from one year to the next.

### Ethics approval

All shark capture and research protocols were approved under Arrêté N° 9524 issued by the Ministère de la Promotion des Langues, de la Culture, de la Communication et de l’Environnement of the French Polynesian government on 30 October 2015 and James Cook University’s Animal Ethics Committee (A2394 and A2769). All applicable international, national, and/or institutional guidelines for the sampling of animals were followed. All the animal procedure were carried out in accordance with ARRIVE guideline.

### Supplementary Information

Supplementary Table S1.

## Data Availability

All data are available in the main text or the supplementary materials. The detail of the microsatellite markers used in this study are available in the supplementary materials.
