# Supplementary material for: Genetic evidence for plastic reproductive philopatry and matrotrophy in blacktip reef sharks (Carcharhinus melanopterus) of the Moorea Island (French Polynesia)
Source: Sci Rep. 2023 Sep 9;13:14913. doi: 10.1038/s41598-023-40140-6 (PMC10492826; doi:10.1038/s41598-023-40140-6)
Supplement: Supplementary file 1 — Supplementary Table S1. [file 41598_2023_40140_MOESM1_ESM.docx]

Supplementary information:

Table S1 - Summary of the 14 microsatellites used in the present study [Ta: annealing temperature, N: Number of scored individuals, Ho: observed heterozygosity, He: expected heterozygosity, k: Number of alleles, Fis: Inbreeding Coefficient (exact test for depart departure from Hardy-Weinberg: *P < 0.05, **P < 0.01, ***P < 0.001 after Bonferroni correction, NS: not significant)]

| Locus | Primer sequence 5’-3’ | Repeat type | Ta | N | H0 | HE | k | Fis | Source |
| --- | --- | --- | --- | --- | --- | --- | --- | --- | --- |
| Cli 107 | F: GGATTCACAACACAGGGAAC  R: CTCATTCTTAGTTGCTCTCG | (GT)14 | 57°C | 230 | 0.447 | 0.448 | 4 | -0.007 NS | Keeney *et al*. 2005 [48] |
| Cli 111 | F: ACTTACGAACTGTTGCTAACTC  R: GGGAGATAAACGACAAATGTG | (GT)28 | 57°C | 230 | 0.855 | 0.786 | 21 | 0.299 NS | Keeney *et al*. 2005 [48] |
| LS20 | F: AAGTCCTGGAGCACAGCCTTCAAG  R: AAACTCCCATTGGAATCGTGG | (AC)8GC(AC)3 | 57°C | 230 | 0.388 | 0.337 | 3 | -0.055 ** | Feldheim *et al*. (2002) [49] |
| Cli103 | F: GCTTCATTCCATGAGAG  R: TTTCTCTGTCCTGGTGTTTC | (GA)5(GA)14(GA)7 | 57°C | 230 | 0.520 | 0.435 | 3 | -0.013* | Keeney *et al*. 2005 [48] |
| LS75 | F: TGTTACTGGGCACTATTATTC  R: GAGGTTATCTTTTCTGTGTAGT | (TC)11(AC)11AG(AC)10 | 57°C | 230 | 0.622 | 0.536 | 3 | 0.013 NS | Feldheim *et al*. (2002) [49] |
| LS32 | F: TTAAGTCAGGCTATTGTGGACTCGT  R: GCTTGCTTTCACACCTACCCATTT | (AC)4(AG)2(AC)7 | 57°C | 230 | 0.496 | 0.408 | 4 | -0.067 NS | Feldheim *et al*. (2002) [49] |
| LS54 | F: TTGGAAACCGTGGAGGTGAA  R: GGGGAAAAAGAACTGGGACTAATCC | (CT)10(CA)8 | 57°C | 230 | 0.426 | 0.359 | 5 | -0.076 NS | Feldheim *et al*. (2002) [49] |
| Cpl 128 | F: GCTGTGATCTTTGCTGATTGAGC  R: GGATGGTGGATTGTGGATTTTG | (CA)13TA(CA)13 | 57°C | 230 | 0.809 | 0.697 | 19 | 0.055 NS | Portnoy *et al.* (2010) [50] |
| Cli 102 | F: GACTGGCTGACCTAACTAAGC  R: ATCCTGTGGTCCTTCTATC | (GA) 9 | 57°C | 230 | 0.428 | 0.443 | 6 | 0.006*** | Keeney *et al*. 2005 [48] |
| Cli 12 | F: TCCCAGTCACATTTACACATGC  R: GGAAGACCATTGAACCCAATC | (GT)9 | 57°C | 230 | 0.348 | 0.525 | 9 | 0.473*** | Keeney *et al*. 2005 [48] |
| Pgl 02 | F: ACCCGACTCGCCAGGATTCACT  R: CCCGAGTCACTCACCGC | (TCC)5TCG(TCC)2 (TCG)2 | 63°C | 230 | 0.595 | 0.518 | 6 | -0.01 NS | Chapman *et al.* (2004) [51] |
| Cs02 | F: GGCTCCATAAAAAAAGTGTTGGTA  R: GGCTCTGTTTAATGTGATGAATGTA | (GT)2(GC)(GT)(GC)(GT)11 | 63°C | 230 | 0.797 | 0.745 | 16 | 0.019 NS | Ovenden *et al.* (2006) [52] |
| Cli 2 | F: CTTTGAGGAAGTTGGTACTGATG  R: GCCACTCTTGTTCTGAATTTTTCCG | (GA)10 | 63°C | 230 | 0.292 | 0.227 | 2 | 0.042 NS | Keeney *et al*. 2005 [48] |
| Cli 108 | F: TCACTGGGTTAGACACTTCC  R: CCACAGTCAGAAAACAAATTG | (GT)12 | 63°C | 230 | 0.748 | 0.637 | 5 | -0.003 NS | Keeney *et al*. 2005 [48] |
